# Medical and social costs after using financial incentives to improve medication adherence: results of a 1 year randomised controlled trial

**DOI:** 10.1186/s13104-018-3747-1

**Published:** 2018-09-10

**Authors:** Ernst L. Noordraven, André I. Wierdsma, Peter Blanken, Anthony F. T. Bloemendaal, Cornelis L. Mulder

**Affiliations:** 1 Dual Diagnosis Center (CDP) Palier, Parnassia Psychiatric Institute, 2552 KS The Hague, The Netherlands; 2 Department of Psychiatry, Erasmus University Medical Center, Epidemiological and Social Psychiatric Research Institute, 3015 CE Rotterdam, The Netherlands; 3 Bavo-Europoort Mental Health Care, 3066 TA Rotterdam, The Netherlands; 4 Parnassia Addiction Research Centre (PARC), Brijder Addiction Treatment, Parnassia Psychiatric Institute, 2553 RJ The Hague, The Netherlands

**Keywords:** Financial incentives, Health care costs, Psychosis, Antipsychotics, Adherence

## Abstract

**Objective:**

Offering a financial incentive (‘Money for Medication’) is effective in improving adherence to treatment with depot antipsychotic medications. We investigated the cost-effectiveness in terms of medical costs and judicial expenses of using financial incentives to improve adherence. The effects of financial incentives on depot medication adherence were evaluated in a randomised controlled trial. Patients in the intervention group received €30 a month over 12 months if antipsychotic depot medication was accepted. The control group received mental health care as usual. For 133 patients outcomes were calculated based on self-reported service use and delinquent behaviour and expressed as standard unit costs to value resource use.

**Results:**

The financial incentive resulted in higher average costs related to mental health care (€449.6 versus €355.7). and lower medical costs related to other healthcare services (€52.0 versus €78.4). Relevant differences in social costs related to delinquent behaviour were not found. Although wide confidence intervals indicate uncertainty, incremental cost-effectiveness ratio’s (ICER) indicate that it costs €2080 for achieving a 20% increase in adherence or €3332 for achieving over 80% adherence. In sum, offering money as financial incentive for increasing compliance did not lead to an overall cost reduction as compared to care as usual.

*Trial registration* NTR2350, 01 June 2010

**Electronic supplementary material:**

The online version of this article (10.1186/s13104-018-3747-1) contains supplementary material, which is available to authorized users.

## Introduction

Adherence to treatment with antipsychotic depot medication is associated with remission from symptoms and improved social outcomes [1]. Yet 25% to 50% of people with schizophrenia are non-adherent to their medication regimen due to a lack of illness insight or side effects [2]. Results of randomized controlled trials suggest that offering a financial incentive (‘Money for Medication’) is effective in improving adherence [3, 4]. However, direct costs increase both because a modest financial incentive is offered over an extended period and because logistical arrangements to distribute money in a community mental health context need to be addressed. On the other hand, adherence to antipsychotic medication may be associated with lower risk of psychiatric hospital admissions and may decrease other health and social care costs. In addition, a decrease of psychotic symptoms may contribute to patients’ quality of life and better social adjustment, which could lower societal costs. However, data on cost-effectiveness of medication adherence-enhancing interventions are rare [5]. And although first economic outcomes for offering financial incentives point in the right direction, effect estimates show wide confidence intervals [6].

Therefore, we investigated the medical and judicial costs after offering financial incentives to achieve better adherence. In a trial studying the effects of financial incentives on depot-adherence and psychosocial outcomes [4], we found a significant improvement in adherence rates, although no effects were found on psychosocial outcomes, including quality of life. Here our focus is on patients’ health care consumption and costs that incurred because of illegal activities. We did not study cost-effectiveness in terms of quality adjusted life years (QALY’s), since the intervention did not affect quality of life, which was a secondary outcome. We investigated the differences in direct medical costs (related to psychiatric treatment), medical costs related to other healthcare services, and judicial costs, between the intervention and control group, and how these costs are related to better antipsychotic medication adherence. To estimate expenditures from a societal perspective, costs were calculated by multiplying resource use with official charge standards.

## Main text

### Methods

#### Medical and judicial costs

The effect of financial incentives on depot medication adherence was evaluated in a randomised controlled trial: 169 patients with a psychotic disorder were randomised to intervention or control groups, stratified by treatment site, sex, comorbid substance-use disorder, and medication compliance [7]. Patients in the control group received mental and primary health care as usual. Patients in the intervention group received the same treatment plus €30 a month over 12-months if antipsychotic depot medication was fully taken. For 35 patients no data were available regarding costs, yet baseline and follow-up proportions of patients using services correspond. Therefore we calculated for 133 (79%) patients direct medical costs and costs related to other healthcare services based on standard unit costs to value resource use at baseline and after 12-months follow-up. Data were collected from the patients’ file, from the depot acceptance registration forms, and from questionnaires that assessed use of healthcare services and delinquent behaviour.

#### Costs related to service use

The Treatment Inventory Cost in psychiatric patients (TiC-P) [8] is a frequently used generic self-report outcome measure in adult patients with a psychiatric diagnosis. Validity of self-report service use is acceptable [9]. The full version of the questionnaire includes health care use, medication, and absence of work or other activities. The items concern the volume of medical consumption and productivity loss over the past 4 weeks. We used the part of the TiC-P that comprises 14 structured questions on contacts within the mental health care sector and contacts with other health services, ranging from general practitioner to homecare. Following the guidelines of the Dutch manual of costing studies in health care [10], total costs were calculated as the sum of the product of reported frequencies and the reference price regarding the type of healthcare use. Mental health care costs were considered as part of treatment related direct costs, whereas other medical consumption was labelled as general medical costs related to other healthcare services. Table 1 summarizes the medical cost items, reference prices, and the number of contacts or hospital days at baseline.

**Table 1:** Service unit costs and average costs per patient at baseline (previous 4 weeks)

	Unit costs €	n (%) patients using service	Average costs per patient (SD)
Medical costs related to psychiatric treatment			
Contact with a caregiver from a regional institute for outpatient mental healthcare	113	150 (89%)	408.3 (509.4)
Contact with a psychiatrist, psychologist or psychotherapist at a private (group) practice	95	16 (10%)	14.1 (66.1)
Contact with a psychiatrist, psychologist or psychotherapist (i.e. outpatient visit in hospital)	95	11 (7%)	12.4 (69.2)
Contact with a clinic for alcohol and drugs	31	2 (1%)	5.5 (66.9)
Participation in a self-help group	58	4 (2%)	3.1 (22.2)
Day- or part-time psychiatric hospital treatment	278	5 (3%)	28.5 (267.9)
Psychiatric hospitalisation	446	6 (4%)	393.2 (2832.8)
Subtotal average sum Excluding hospitalisation		169 (100%)	901.4 (2982.5) 508.2 (682.8)
Medical costs related to other healthcare services			
Contact with a general practitioner	33	44 (26%)	13.1 (25.8)
Contact with a company doctor	33	2 (1%)	0.4 (3.6)
Contact with a medical specialist (i.e. outpatient visit in hospital)	92	17 (10%)	21.8 (102.0)
Contact with a physiotherapist	33	3 (2%)	1.2 (10.7)
Contact with a social worker	65	33 (20%)	37.7 (125.2)
Home care	20	17 (10%)	13.4 (47.5)
Contact with an alternative healer	51	2 (1%)	1.8 (17.5)
Day- or part-time treatment	278	–	–
Other hospital^a^	170	–	–
Hospitalisation	446	3 (2%)	39.6 (310.9)
Subtotal average sum Excluding hospitalisation		169 (100%)	91.2 (337.5) 54.7 (127.6)
Total medical costs			992.6 (3008.8)
Total costs, excluding hospitalisation			559.8 (702.1)

^a^ Other than a general hospital, an academic hospital, or a rehabilitation center

#### Costs related to delinquency

The Self-Reported Delinquency questionnaire (SRD) provides an account of a wide range of illegal acts and has been widely used, although item difficulty varies across subgroups [11]. We copied the questionnaire from the Dutch version of the INternational CAnnabis Need of Treatment study (INCANT) [12–14]. The SRD questionnaire examines the frequency of minor delinquent acts (e.g. vandalism) and criminal acts (e.g. armed robbery). Patients were asked to report on the number of times the specified delinquent behaviour was performed in the last 4 weeks. Contrary to health care contacts, types of delinquency have no generally accepted reference costs. However, Goorden et al. [15] estimated costs based on annual judicial expenses and the number of registered crimes and violations broken down into categories comparable to categories used in the SRD. We followed this approach to differentiate costs linked to the SRD items; the unit prices were multiplied by the reported frequency of the specific delinquent behaviour and summed to obtain an estimate of the total delinquency costs. Additional file 1: Appendix S1 shows the list of types of delinquent behaviour, unit prices, and the reported frequencies at baseline.

#### Statistical analysis

Medical costs are typically characterized by an asymmetry of the distribution because some patients have minimal costs or specific standard cost amounts and other patients may have disproportionately high costs. Generalized linear models using a log-gamma distribution, have been suggested to account for this kind of highly skewed data [16]. We used the GenLin procedure in SPSS version 21 to model differences in direct mental healthcare costs, medical costs related to other healthcare services, and judicial costs between the intervention and control groups. Means and standard deviations are reported to describe the costs per category of service use and type of delinquency and to illustrate the asymmetry of cost data. Both medical and judicial total costs are dominated by items that are infrequent but have relatively high unit prices. Table 1 shows that an important part of the average medical costs per patient comes from only a few patients who were hospitalised. In Additional file 1: Appendix S1 the social costs of robbery stand out. Therefore, we looked at differences in the sum of costs both with and without including hospitalisation costs. Multivariable analysis focussed on the main effect of the intervention on medical and judicial costs, adjusting for stratification variables (i.e. gender, baseline compliance and substance use). Statistical significance of the regression coefficient was tested using the Wald-test and a conventional .05 significance level. In addition, an incremental cost-effectiveness ratio (ICER) was calculated by dividing the incremental total costs per year by the incremental effects and creating a bootstrapped 95% confidence interval based on 1000 replications. First, we considered the incremental costs of achieving a 20% increase in adherence following Henderson et al. [6]. Secondly, we calculated the incremental costs of achieving ‘good’ adherence i.e., taking at least 80% of the prescribed depot medications over the 12-month intervention period, since this cut-off has been recommended by expert consensus guidelines [17].

### Results

At baseline, between groups differences were negligible. For a detailed account of baseline patient characteristics see Noordraven et al. [4]. An adherence difference of 14.9% (95% CI 8.9%, 20.9%) was found for the medication possession ratio, and the difference in the proportion of patients achieving good (≥ 80%) adherence levels was 33.1% (95% CI 20.2% to 45.4%) in favour of in the intervention group [4]. This result is reflected in higher costs related to psychiatric treatment at 12-months follow-up in the intervention condition compared to care as usual (€1062.9 vs. €788.8). However, regression analysis controlling for stratification variables indicated a statistically insignificant difference in total medical costs between the intervention and control group (€1592.5 vs. €1272.8; B = .517, SE = .282, p = .067). Table 2 shows that this difference is due to costs of psychiatric hospitalisation, not as much to more frequent regular contacts with outpatient mental health care excluding hospitalisations (€484.4 vs. €432.5; B = .251, SE = .206, p = .222).

**Table 2:** Service costs at 12 months follow-up (previous 4 weeks)

	Intervention group n (%) patients	Average costs (SD)	Control group n (%) patients	Average costs (SD)
Medical costs related to psychiatric treatment				
Contact with a caregiver from a regional institute for outpatient mental healthcare	58 (91%)	410.1 (532.5)	60 (87%)	269.9 (361.1)
Contact with a psychiatrist, psychologist or psychotherapist at a private (group) practice	5 (8%)	12.1 (55.3)	17 (25%)	33.5 (78.3)
Contact with a psychiatrist, psychologist or psychotherapist (i.e. outpatient visit in hospital)	2 (3%)	2.9 (16.7)	3 (4%)	4.1 (19.5)
Contact with a clinic for alcohol and drugs	–	–	1 (1%)	0.9 (7.5)
Participation in a self-help group	3 (5%)	17.2 (93.4)	1 (1%)	1.7 (13.9)
Day- or part-time psychiatric hospital treatment	–	–	1 (1%)	4.0 (33.5)
Psychiatric hospitalisation	3 (5%)	613.3 (2788.7)	3 (4%)	433.1 (2284.4)
Intervention costs financial incentives	64 (100%)	28.6 (3.2)	0 (0%)	0 (0.0)
Subtotal average sum Excluding hospitalisation		1062.9 (3031.5) 449.6 (530.4)		788.8 (2379.3) 355.7 (463.9
Medical costs related to other healthcare services				
Contact with a general practitioner	16 (25%)	8.8 (15.8)	22 (32%)	15.5 (28.2)
Contact with a company doctor	1 (1%)	0.5 (4.1)	1 (1%)	0.5 (4.0)
Contact with a medical specialist (i.e. outpatient visit in hospital)	3 (5%)	4.3 (19.6)	9 (13%)	14.7 (40.6)
Contact with a physiotherapist	2 (3%)	4.6 (33.2)	2 (3%)	3.3 (21.3)
Contact with a social worker	9 (14%)	20.6 (64.8)	12 (17%)	50.6 (241.9)
Home care	5 (8%)	16.6 (60.9)	5 (7%)	10.7 (43.2)
Contact with an alternative healer	–	–	–	–
Day- or part-time treatment	–	–	1 (1%)	32.2 (267.7)
Hospitalisation	4 (6%)	494.8 (2246.4)	3 (4%)	407.2 (2263.5)
Subtotal average sum Excluding hospitalisation		529.6 (2241.7) 52.0 (117.8)		484.0 (2266.6) 78.4 (278.4)
Total costs Excluding hospitalization	64 (100%)	1592.5 (3700.7) 484.4 (538.9)	69 (100%)	1272.8 (3223.7) 432.5 (536.1)

In the intervention group average medical costs related to other healthcare services were somewhat higher compared to the control group (€529.6 versus €484.0), but lower after excluding hospitalisation (€52.0 versus €78.4). Fewer patients in the Money-for-Medication program visited their GP, a medical specialist, or social worker. This effect was in the expected direction but small (statistical models did not adequately converge).

Additional file 1: Appendix S1 illustrates that delinquent behaviour is not very common among patients with psychotic disorder. Minor offences are most frequently reported but less than 6% of patients are involved in shoplifting incidents or buying and selling stolen goods. At 12-months follow-up very few patients reported delinquent behaviour (Table 3) and only small differences in related social costs were found comparing the intervention group and the control group (€248.4 vs. €229.3; B = .607, SE = .420, p = .149). During the 18-month follow up period, results remained comparable both for the healthcare (Additional file 2: Appendix S2) and judicial related costs (Additional file 3: Appendix S3).

**Table 3:** Delinquent behaviour costs at 12 months follow-up (previous 4 weeks)

	Intervention Group n (%) patients	Average costs (SD)	Control Group n (%) patients	Average costs (SD)
Damaged a vehicle	–	–	–	–
Damaged public objects	–	–	–	–
Besmirched something	–	–	–	–
Arson	–	–	–	–
Changed price labels in a shop	–	–	–	–
Shoplifting	1 (1%)	28.8 (237.7)	–	–
Stole something at work	–	–	–	–
Stole a bicycle or scooter	–	–	1 (1%)	148.5 (1206.3)
Stole something of a car	–	–	–	–
Buying stolen goods	3 (4%)	75.9 (460.0)	–	–
Soled something stolen	1 (1%)	24.9 (205.4)	1 (1%)	25.7 (208.5)
Stole something out of a car	1 (1%)	28.8 (237.7)	–	–
Cartheft	–	–	–	
Burglary	–	–	–	–
Pickpocketing	1 (1%)	28.8 (237.7)	–	–
Robbery	–	–	–	–
Agressive behavior	–	–	–	–
Violent behavior	–	–	1 (1%)	55.1 (447.8)
Armed violence	1 (1%)	62.3 (513.4)	–	–
Total	64 (100%)	248.4 (856.2)	69 (100%)	229.3 (1477.4)

On average, the maximum of 30 euro extra cost item as financial incentive per patient per month constitutes about 3% of average total mental healthcare costs (€1062) and less than 7% of outpatient medical costs (€449). Extrapolating costs, excluding hospitalisation, in the previous 4 weeks at 12 months follow-up to total costs per patient per year, averaged to €9273 (SD 13512) in the Money-for-Medication group and to €7900 (SD 19089) in the care-as-usual group. Incremental total costs were €2080 (95% CI − 37972 to 34811) for achieving a 20% increase in adherence and €3332 (95% CI − 22675 to 28128) for taking at least 80% of the prescribed depot medications over the 12-month intervention period.

### Discussion

Providing a financial incentive to improve adherence to depot medication in psychotic patients resulted in higher average costs directly related to mental health care and lower costs related to other health care services. Relevant differences in social costs related to delinquent behaviour were not found.

An increase in medication compliance was reflected in mental healthcare costs, which were higher in the Money-for-Medication group compared to the control group. In contrast, medical costs related to other health care services were somewhat lower in the intervention group. Effects in terms of medical costs were in the expected direction but differences between the intervention and control group were not statistically significant. Social costs related to delinquency concerned few patients and only minor and non-significant differences were found comparing the intervention and control group.

Intervention costs are low considering a maximum financial incentive of 360 euro per patient per year. Currently no threshold values are available for the ICER-values in the range of €2000 for achieving a 20% increase in adherence, and just over €3000 for ‘good’ (80% or higher) medication adherence. Interestingly, these figures are in line with the results of Henderson et al. [6], which estimated these costs in the range of €1144 and €3400 respectively. The ‘Money for Medication’ study supports these results and is the first trial within the Netherlands and the second and largest trial worldwide, which makes it and an important replication study. In sum, this suggests that we may be able to increase compliance with depot medication to an appropriate level when we are willing to invest extra. However, incremental cost-effectiveness ratio’s (ICER) showed wide confidence intervals indicating a high level of uncertainty.

### Conclusions

Financial incentives are effective in improving treatment adherence in patients with psychotic disorder. However, offering money as financial incentive for increasing compliance did not lead to an overall cost reduction as compared to care as usual. Perhaps that financial benefits of M4M in terms of reductions in medical costs might become manifest only after a longer period of time. Therefore, future studies using longer intervention and follow-up periods are needed to investigate cost-effectiveness also with respect to quality of life.

## Limitations

Medical and judicial cost items were patient reported over a 4 weeks’ time span which may reduce memory bias, but may not adequately reflect variability in the level of health service use or delinquent behaviour in our 12-month study period. Also, the national reference costs per health care contact or type of delinquency were crude estimates of the true mental healthcare cost, medical costs related to other health care services, and social costs.

The Self-Reported Delinquency questionnaire originally was aimed at adolescents and may be less suited for mapping delinquent behaviour in psychiatric patients, which may explain the low frequency of reported delinquent behaviour. In addition, we maybe overestimated the amount of criminal activities within a non-forensic patient setting; patients with chronic psychiatric diseases are not necessarily involved in criminal activities.

During this study, frequency of other social parameters (e.g. participation in volunteer work) were not assessed and the invested time per patient to arrange appointments for proving depot medication was not monitored, so it remains unclear whether implementing M4M did actually save or cost extra time. Furthermore, our study was underpowered for the analysis of highly skewed cost data, resulting in wide bootstrapped confidence intervals for incremental cost-effectiveness ratios.

## Additional files

**Additional file 1: Appendix S1.** Judicial unit prices and baseline costs. The file includes a table with the judicial unit prices and costs at baseline for all patients.

**Additional file 2: Appendix S2.** Service costs 18 months. The file includes a table with the follow-up data for the service costs at 18 months.

**Additional file 3: Appendix S3.** Judicial costs 18 months. The file includes a table with the follow-up data for the service costs at 18 months.

## Abbreviations

M4M: Money for Medication
SRD: Self-Reported Delinquency Scale
ICER: incremental cost-effectiveness ratio
QALY´s: quality adjusted life years
TiC-P: Treatment Inventory Cost in psychiatric patients
INCANT: INternational CAnnabis Need of Treatment
