# Supplementary material for: Medical and social costs after using financial incentives to improve medication adherence: results of a 1 year randomised controlled trial
Source: BMC Res Notes. 2018 Sep 10;11:655. doi: 10.1186/s13104-018-3747-1 (PMC6131864; doi:10.1186/s13104-018-3747-1)
Supplement: Supplementary file 1 — Additional file 1: Appendix S1. Judicial unit prices and baseline costs. The file includes a table with the judicial unit prices and costs at baseline for all patients. [file 13104_2018_3747_MOESM1_ESM.docx]

Appendix S1. Unit costs to value delinquent behaviour and average costs per patient at baseline (previous four weeks)

|  | Unit costs ^a^ € | n (%) patients | Average Costs per patient (SD) |
| --- | --- | --- | --- |
| Damaged a vehicle | 1910 | 3 (2%) | 34.5 (255.2) |
| Damaged public objects ^b^ | 733 | 3 (2%) | 8.9 (80.5) |
| Besmirched something ^b^ | 733 | 3 (2%) | 8.9 (80.5) |
| Arson | 1449 | - | - |
| Changed price labels in a shop ^b^ | 549 | 1 (1%) | 3.3 (42.6) |
| Shoplifting | 1960 | 10 (6%) | 167.3 (1027.6) |
| Stole something at work | 1960 | - | - |
| Stole a bicycle or scooter | 1960 | 1 (1%) | 11.8 (152.1) |
| Stole something of a car | 1910 | - | - |
| Buying stolen goods | 1694 | 4 (2%) | 81.6 (584.1) |
| Soled something stolen | 1694 | 5 (3%) | 51.0 (290.4) |
| Stole something out of a car | 1960 | - | - |
| Car theft ^c^ | 5000 | - | - |
| Burglary ^d^ | 4667 | 1 (1%) | 28.1 (362.2) |
| Pickpocketing | 1960 | 1 (1%) | 11.8 (152.1) |
| Robbery | 20.939 | 2 (1%) | 252.3 (2291.4) |
| Aggressive behavior | 1819 | 2 (1%) | 11.0 (141.6) |
| Violent behavior | 4234 | 2 (1%) | 76.5 (733.0) |
| Armed violence | 4234 | - | - |
| Total |  | 169 (100%) | 744.9 (3615.4) |

**^a^** Unit costs based on Goorden et al (2016) unless otherwise specified; **^b^** Groot et al. (2007);

^c^ van Ours & Vollaard (2013); ^d^ Vollaard (2010)
