# Supplementary material for: Medical and social costs after using financial incentives to improve medication adherence: results of a 1 year randomised controlled trial
Source: BMC Res Notes. 2018 Sep 10;11:655. doi: 10.1186/s13104-018-3747-1 (PMC6131864; doi:10.1186/s13104-018-3747-1)
Supplement: Supplementary file 2 — Additional file 2: Appendix S2. Service costs 18 months. The file includes a table with the follow-up data for the service costs at 18 months. [file 13104_2018_3747_MOESM2_ESM.docx]

Appendix S2. Service costs at 18 months follow-up (previous four weeks)

|  | Intervention Group n (%) patients | Average costs  (SD) | Control Group n (%) patients | Average costs  (SD) |
| --- | --- | --- | --- | --- |
| Medical costs related to psychiatric treatment |  |  |  |  |
| Contact with a caregiver from a regional institute for outpatient mental healthcare | 45 (85%) | 421.6 (638.1) | 47 (89%) | 268.6 (203.4) |
| Contact with a psychiatrist, psychologist or psychotherapist at a private (group) practice | 5 (9%) | 17.9 (64.7) | 6 (11%) | 19.7 (65.5) |
| Contact with a psychiatrist, psychologist or psychotherapist (i.e. outpatient visit in hospital) | 4 (8%) | 11.0 (55.3) | 0 (0%) | - |
| Contact with a clinic for alcohol and drugs | 2 (4%) | 5.3 (34.2) | 1 (2%) | 0.6 (4.3) |
| Participation in a self-help group | 1 (2%) | (93.4) | 1 (2%) | (13.9) |
| Day- or part-time psychiatric hospital treatment | - | - | - | - |
| Psychiatric hospitalisation | 2 (3%) | 429.2 (2223.1) | (0%) | - |
| Intervention costs financial incentives | - | - | - | - |
| Subtotal average sum  excluding hospitalisation |  | **903.5 (2241.2)**  474.3 (704.1) |  | **303.5 (217.2)**  303.5 (217.2) |
| Medical costs related to other healthcare services |  |  |  |  |
| Contact with a general practitioner | 19 (36%) | 16.2 (33.2) | 14 (26%) | 9.9 (17.8) |
| Contact with a company doctor | 1 (2%) | 0.6 (4.5) | 0 (0%) | - |
| Contact with a medical specialist (i.e. outpatient visit in hospital) | 5 (9%) | 10.4 (34.5) | 4 (8%) | (55.0) |
| Contact with a physiotherapist | 1 (2%) | 5.0 (36.3) | 0 (0%) | - |
| Contact with a social worker | 8 (15%) | 24.5 (66.5) | 5 (9%) | 13.5 (48.3) |
| Home care | 6 (11%) | 39.2 (162.2) | 8 (15%) | 16.2 (47.1) |
| Contact with an alternative healer | 1 (2%) | 0 | 1 (2%) | 0 |
| Day- or part-time treatment | - | - | - | - |
| Hospitalisation | 2 (3%) | 732.1 (5267.8) | 1 (1%) | 16.8 (122.5) |
| Subtotal average sum  excluding hospitalisation |  | **802.9 (5261.0)**  73.0 (187.1) |  | **58.6 (178.1)**  42.9 (95.3) |
|  |  |  |  |  |
| Total costs  excluding hospitalization | 53 (100%) | **1706.4 (5639.3)**  545.1 (757.0) | 53 (100%) | **362.2 (285.1)**  345.3 (230.7) |
