# Supplementary material for: Medical and social costs after using financial incentives to improve medication adherence: results of a 1 year randomised controlled trial
Source: BMC Res Notes. 2018 Sep 10;11:655. doi: 10.1186/s13104-018-3747-1 (PMC6131864; doi:10.1186/s13104-018-3747-1)
Supplement: Supplementary file 3 — Additional file 3: Appendix S3. Judicial costs 18 months. The file includes a table with the follow-up data for the service costs at 18 months. [file 13104_2018_3747_MOESM3_ESM.docx]

Appendix S3. Delinquent behaviour costs at 18 months follow-up (previous four weeks)

|  | Intervention Group n (%)  patients | Average costs  (SD) | Control Group n (%) patients | Average costs  (SD) |
| --- | --- | --- | --- | --- |
| Damaged a vehicle | - | - | - | - |
| Damaged public objects | - | - | - | - |
| Besmirched something | 1 (2%) | 12.0 (93.9) | - | - |
| Arson | - | - | - | - |
| Changed price labels in a shop | - | - | - | - |
| Shoplifting | 2 (3%) | 64.3 (351.9) | - | - |
| Stole something at work | - | - | - | - |
| Stole a bicycle or scooter | 1 (2%) | 32.1 (251.0) | 1 (2%) | 38.4 (274.5) |
| Stole something of a car | - | - | 1 (2%) | 37.5 (267.5) |
| Buying stolen goods | 1 (2%) | 27.8 (216.9) | 1 (2%) | 33.2 (237.2) |
| Soled something stolen | 1 (2%) | 27.8 (216.9) | 1 (2%) | 33.2 (237.2) |
| Stole something out of a car | 1 (2%) | 32.1 (251.0) | - | - |
| Cartheft | - | - | - |  |
| Burglary | 1 (2%) | 76.5 (597.5) | - | - |
| Pickpocketing | - | - | - | - |
| Robbery | - | - | - | - |
| Agressive behavior | 1 (2%) | 59.6 (465.8) | - | - |
| Violent behavior | 1 (2%) | 69.4 (542.1) | - | - |
| Armed violence | - | - | - | - |
| Total | 61 (100%) | 401.6 (1408.6) | 51 (100%) | 104.9 (748.9) |
